# The Effect of an Aquatic Neuromuscular Exercise Program on Pain Alleviation and Functional Performance in Cases of Chronic Achilles Tendinopathy Among Overweight Middle-Aged Women

**DOI:** 10.7759/cureus.89304

**Published:** 2025-08-03

**Authors:** Sawani Aphale, Sandeep Shinde, Harshal Y Kale, Manoj P Ambali

**Affiliations:** 1 Department of Musculoskeletal Sciences, Krishna College of Physiotherapy, Krishna Vishwa Vidyapeeth (Deemed to Be University), Karad, IND; 2 Department of Critical Care Medicine, Krishna Institute of Medical Sciences, Krishna Vishwa Vidyapeeth (Deemed to Be University), Karad, IND; 3 Department of Anatomy, Krishna Institute of Medical Sciences, Krishna Vishwa Vidyapeeth (Deemed to Be University), Karad, IND

**Keywords:** achilles tendon, mobility limitation, pain measurement, range of motion, rehabilitation

## Abstract

Background: Chronic Achilles tendinopathy is characterized by persistent pain, swelling, and functional limitations, particularly in overweight middle-aged women. This demographic is predisposed to tendon degeneration due to biomechanical overload and hormonal fluctuations.

Objective: This study aimed to compare the effectiveness of an aquatic neuromuscular exercise program vs. a conventional land-based exercise program in overweight middle-aged women with chronic Achilles tendinopathy, using pain intensity (visual analogue scale), ankle dorsiflexion range of motion (weight-bearing lunge test, WBLT), and functional performance (patient-specific functional scale, PSFS).

Methods: A randomized controlled study was conducted on 102 overweight women aged 40-50 years (body mass index 25-29.9 kg/m²), clinically diagnosed with chronic Achilles tendinopathy. Participants were randomly assigned to Group A (aquatic neuromuscular program) or Group B (conventional exercise therapy). Both groups underwent supervised exercise sessions four times weekly for four weeks, with progressive intensity. Outcome measures included the visual analog scale for pain, the WBLT for ankle dorsiflexion, and the PSFS. Data were analyzed using paired and unpaired t-tests. Mean differences, standard deviations, p values, and effect sizes were reported.

Results: Group A showed superior improvements across all outcome measures. Pain during activity reduced from 5.96 ± 1.26 to 1.36 ± 0.88 (effect size: 4.23), dorsiflexion improved from 15.24 ± 1.47° to 27.34 ± 1.77° (effect size: 7.44), and PSFS scores improved from 15.21 ± 2.86 to 28.65 ± 2.74 (effect size: 4.80). Group B showed statistically significant but comparatively smaller gains. Statistical significance (p < 0.0001) was observed in all outcomes.

Conclusion: This four-week aquatic neuromuscular exercise program proved superior to conventional exercises in reducing pain, improving ankle dorsiflexion, and enhancing functional performance in overweight middle-aged women with chronic Achilles tendinopathy. These findings support the use of aquatic-based neuromuscular rehabilitation as a clinically beneficial and low-impact intervention for this population.

## Introduction

Achilles tendinopathy is a common overuse injury of the lower limb, characterized by localized pain, tendon swelling, and reduced function during activities such as walking and running. Histopathological studies reveal a disrupted tendon matrix marked by collagen disorganization, increased cellularity, and neovascular ingrowth, indicative of chronic degeneration rather than acute inflammation. The condition occurs in both athletic and sedentary populations, particularly in middle-aged individuals. Intrinsic factors such as biomechanical abnormalities, elevated body mass index (BMI), and hormonal fluctuations contribute to increased tendon vulnerability [1].

The calcaneal tendon, commonly referred to as the Achilles tendon, is the thickest and strongest tendon in the human body. It is anatomically located in the posterior lower limb and connects the gastrocnemius and soleus muscles, collectively known as the calf muscles, to the calcaneal tuberosity of the heel bone [2,3]. The soleus and gastrocnemius contribute to gait, with the soleus primarily stabilizing the leg over the foot. Tendon innervation is chiefly derived from the sural and posterior tibial nerves [4]. It functions to transmit and modulate muscular force, thereby preventing injury. Elevated BMI increases mechanical stress on the tendon, promoting pain development [5].

As an overuse injury, Achilles tendinopathy typically results from repetitive stress due to the tendon’s involvement in daily movements. Among overweight middle-aged women, age-related hormonal changes, increased BMI, reduced bone mineral density, and diminished muscular flexibility frequently led to musculoskeletal discomfort. During the perimenopausal period, levels of hormones such as estrogen, progesterone, and testosterone begin to decline. Additionally, changes in growth hormone and thyroid hormone levels negatively affect musculoskeletal health. These hormonal fluctuations directly impact collagen synthesis, tendon development, and the mechanical integrity of the tendon. Notably, estrogen plays a pivotal role in maintaining musculoskeletal health; a reduction in its levels has been associated with impaired muscle function [6].

Age-related declines in calcium levels further exacerbate bone demineralization, manifesting as muscle cramps and generalized pain, thereby intensifying perimenopausal symptoms [7]. A marked decline in physical activity is commonly observed among aging women, attributed to reduced muscle strength, flexibility, and endurance [8].

Gait adaptations are frequently observed in women experiencing pain in the calcaneal tendon. To reduce pain, they often decrease ankle dorsiflexion during ambulation. Over time, this compensation may restrict mobility and shift the center of gravity anteriorly, thereby increasing the risk of falls [9]. A growing body of evidence suggests that reduced ankle dorsiflexion, especially when assessed in weight-bearing positions, is associated with increased symptom severity in individuals with mid-portion Achilles tendinopathy. A cross-sectional study by Scattone Silva et al. found no significant side-to-side difference in dorsiflexion range. However, they identified a strong association between limited dorsiflexion and more pronounced clinical symptoms in the affected limb [10]. Calf muscle dysfunction, particularly in physically inactive women, raises the risk of developing Achilles tendinopathy. Reduced flexibility in the calf muscles places additional stress on the calcaneal tendon, increasing the likelihood of tendinous strain and muscle-related complications [11].

A previous study examined neuromuscular control in individuals with Achilles tendinopathy and revealed altered motor control of the triceps surae, comprising the gastrocnemius and soleus. These muscles, responsible for plantarflexion, demonstrated dysfunctional activation patterns, including delayed recruitment, imbalanced muscle firing, and inefficient load distribution. Such neuromuscular deficits may increase mechanical stress on the tendon, impair movement efficiency, and potentially delay recovery or lead to symptom recurrence [12].

Neuromuscular exercise programs aim to restore dynamic joint stability by improving muscle coordination, joint proprioception, and functional strength. A previous study investigating proprioceptive and neuromuscular training for rehabilitation of upper and lower limb orthopedic injuries had utilized a wide range of exercises. Common interventions include balance training on stable and unstable surfaces, often incorporating perturbations to challenge postural control. Some protocols further integrate resistance training, plyometrics, and agility drills as part of a multimodal neuromuscular rehabilitation approach [13].

Aquatic therapy, or hydrotherapy, is a well-established intervention for managing musculoskeletal disorders. It involves performing therapeutic exercises in a temperature-regulated pool, providing a supportive, low-impact environment ideal for individuals experiencing joint discomfort, muscle weakness, or restricted mobility. The unique properties of water, such as buoyancy, resistance, and hydrostatic pressure, help alleviate pain, enhance joint range of motion, and support overall functional recovery [14].

Aquatic therapy offers a low-impact environment that reduces tendon loading while facilitating neuromuscular activation, making it particularly suitable for overweight middle-aged women. The aquatic environment provides the offloading effect, which minimizes mechanical stress on the Achilles tendon while allowing for safe and progressive activation of the calf musculature. Additionally, the inherent resistance of water facilitates strength, proprioception, and joint mobility without exacerbating symptoms. This population often presents with biomechanical and hormonal factors that heighten tendon vulnerability, yet remains underrepresented in rehabilitation research.

This neuromuscular exercise program integrated balance, proprioception, dynamic joint stability, and functional strength within a water-based environment. Therefore, the study was undertaken to determine and compare the effects of an aquatic neuromuscular program with those of a conventional exercise program in managing chronic Achilles tendinopathy in middle-aged women.

## Materials and methods

This was a comparative study conducted at Krishna Vishwa Vidyapeeth (Deemed to Be University), Karad. Using the following formula [15], the sample size was estimated to be 102, where prevalence (p) was 7% [16], q (100 - p) was 93%, and allowable error (L) was 5.

$n=\frac{4pq}{L^{2}}$

It was a single-blinded study where participants were blinded. Participants were randomly allocated to two intervention groups using a simple randomization technique. Allocation was done using the envelope method, where two envelopes labeled as Group A and Group B were prepared. Before the start of the intervention, each participant was instructed to pick one envelope, which determined their group assignment. This method ensured an impartial and unbiased distribution of participants across both groups.

All 102 participants fulfilled the inclusion criteria, which involved a sudden onset of pain in the lower posterior leg, approximately 2-4 cm above the heel, beginning within the past six months. The discomfort typically develops after a recent increase in physical activity, such as prolonged standing or walking. The pain was described as sharp during activity and tended to subside with rest; however, there was noticeable stiffness and soreness during the first few steps in the morning. Participants clinically diagnosed with Achilles tendinopathy, aged between 40 and 50 years, and having a BMI of 25-29.9 kg/m² were included. Individuals were excluded if they had a known history of osteoarthritis of the hip, knee, or ankle; diabetes mellitus; peripheral neuropathy; prior lower limb trauma; foot disorders; peripheral arterial disease; deep vein thrombosis; or if they had undergone surgical interventions involving the hip, knee, or ankle within the past 10 years.

This study had Institutional Ethical Committee approval, and it was conducted in Karad. Participants were selected based on the inclusion and exclusion criteria and were informed about the study procedure, intervention details, and possible benefits. Written informed consent was obtained before the initiation of treatment. Demographic details, including name, age, BMI, and lifestyle, were recorded for each participant before beginning the intervention. To ensure the Achilles and calcaneal tendons were intact, the Thompson test was performed on all subjects. In this test, the participant lay prone with the foot hanging off the edge of the table or with the knee bent at 90°. The examiner then squeezed the gastrocnemius-soleus complex (calf muscles). A normal response would be plantarflexion of the ankle, indicating no rupture of the Achilles or calcaneal tendon [17].

Outcome measures

Primary Outcome Measures

Visual analog scale: Pain intensity was assessed using the visual analog scale (VAS), a 10-cm horizontal line anchored by two descriptors: “0” indicating no pain and “10” indicating the worst imaginable pain. Participants were instructed to mark a point along the line that best represented their current pain level. A higher score corresponded to a greater perceived intensity of pain [18,19].

Patient-specific functional scale: Functional limitations were measured using the patient-specific functional scale (PSFS), a self-reported outcome tool that allows participants to identify and rate activities they find difficult due to their current condition. At the start of the assessment, each participant listed three daily activities they were having difficulty performing. They were then asked to rate their ability to perform each activity on an 11-point scale, where 0 indicated complete inability and 10 indicated full ability to perform the activity. The PSFS, developed by Stratford et al., is freely available for noncommercial use in clinical and research settings without the need for prior permission [20].

Secondary Outcome Measures

Weight-bearing lunge test: Ankle dorsiflexion was evaluated using the weight-bearing lunge test (WBLT), a reliable and clinically valid tool for assessing range of motion in a functional, weight-bearing position. In this test, the participant stood facing a wall and attempted to touch their knee to the wall while keeping the heel firmly grounded. The foot was gradually moved backward until the maximum distance was reached, at which the knee could still contact the wall without lifting the heel. The distance from the great toe to the wall or the angle of tibial inclination was then measured. For angular measurement, the fulcrum of the goniometer was placed over the lateral aspect of the lateral malleolus. The stationary arm was aligned parallel to the lateral border of the fifth metatarsal, and the movable arm was aligned with the lateral midline of the fibula. A dorsiflexion angle of less than 20° was considered indicative of shortened calf muscle length [21].

Before starting the intervention, participants received a detailed demonstration of the full exercise protocol relevant to their group. This was reinforced with a printed “What to Do” checklist, which covered key instructions for posture, breathing, and safety. Physiotherapists clarified the purpose of each movement and ensured that participants were confident in performing the exercises correctly. Safety precautions were strictly maintained throughout the intervention. These included supervised sessions by trained physiotherapists, the use of flotation belts and supports in the aquatic environment, regular monitoring of vital signs, and maintenance of optimal pool temperature. Any signs of discomfort or fatigue were immediately addressed, and no adverse events were reported during the course of the study.

All outcome measures were assessed at baseline (before the start of the intervention) and immediately following the completion of the four-week exercise program. Both Group A and Group B underwent structured exercise intervention administered frequency (four times per week for four weeks, and each week, the exercises were progressively modified in terms of intensity and complexity). Each session included a warm-up, targeted exercise set, and cool-down phase. A neuromuscular exercise program performed in an aquatic environment comprises a structured set of interventions designed to improve proprioception, balance, and dynamic joint stability [22,23]. The aquatic group utilized buoyancy and resistance through water-based tools and movements such as toe and heel raises, lunges, stair walking, and dynamic balance tasks, while the land-based group followed traditional strength and mobility exercises. Group A followed an aquatic neuromuscular program, while Group B participated in a conventional land-based exercise program that included dynamic stretching, strengthening, and mobility exercises, as detailed in Table 1.

**Table 1:** Exercise intervention for both the groups

Week	Phases of exercise training	Group A		Group B	
		Exercise protocol (aquatic neuromuscular program)	Repetitions/duration	Exercise protocol (land-based exercises)	Repetitions/duration
For all sessions	Warm up	Deep breathing exercises, including thoracic expansion exercises and pursed lip breathing	3 minutes	The same as group A	
Week 1	Exercise training	Aqua toe and heel raise (neutral, in, out), holding pool wall	10 times each	Ankle toe movements	10 times
		Aquatic lunges	10 sec hold × 3 sets	Heel slides	10 times
		Aquatic ankle-toe movements	10 times	Bilateral heel raises (neutral, heel in and out)	10 times each
		Heel slides on pool floor (seated or supported)	10 times	Heel slides	10 times
		Toe walking forward in water	20 meters × 1 set	Toe walking	20 meters × 1 set
		Heel walking backward in water	20 meters × 1 set	Heel walking	20 meters × 1 set
Week 2	Exercise training	Aqua heel raises (neutral, in, out) at the wall	10 times × 2 sets	Dynamic calf stretching	10 seconds hold × 3 sets
		Aquatic eccentric heel drops (pool steps, knees straight and bent)	10 times × 2 sets	Bilateral heel raises (neutral, heel in and out)	10 times × 2 sets
		Side-stepping in water	10 times × 1 set	Unilateral heel raises	10 times × 2 sets
		Toe walking forward in water	20 meters × 1 set	Toe walking	20 meters × 1 set
		Heel walking backward in water	20 meters × 1 set	Heel walking	20 meters × 1 set
Week 3	Exercise training	Seated heel raises using aquatic weight cuffs	10 times × 1 set	Dynamic calf stretching	15 seconds hold × 3 sets
		Step-ups on submerged pool stairs	10 stairs × 1 set	Stair walking (0.5 kg weight)	10 stairs × 2 set
		Side stepping in water	20 reps × 1 set	Toe walking	20 meters × 2 set
		Toe walking	20 meters × 2 set	Heel walking	20 meters × 2 set
		Heel walking	20 meters × 2 set	Seated heel raises (0.5 kg weight)	10 times × 1 set
		Weight shifts	10 times × 2 sets	Weight shifts	10 times × 2 sets
Week 4	Exercise training	Ankle dorsiflexion and plantarflexion using aqua resistance fins or paddles	10 times × 2 sets	Ankle dorsiflexion and plantarflexion using TheraBand (The Hygenic Corporation, Akron, OH)	10 times × 2 sets
		Heel raises with aquatic weight cuffs	10 times × 2 set	Heel raises (0.5 kg weight)	10 times × 2 sets
		Stair walking in water by using aquatic weight cuffs	10 stairs × 1 set	Stair walking (1 kg weight)	10 stairs × 2 set
		Lateral hops (with flotation belt support if needed)	10 reps × 1 set	Lateral hops	10 times × 1 set
		Single-leg standing	10 seconds hold × 3 sets	Single leg standing	10 seconds hold × 3 sets
		Light jogging with high knees in water	3 minutes	Light jogging	3 minutes
For all sessions	Cool down	Tendoachilles stretching	10 seconds hold × 2 sets	The same as group A	
		Relaxation regime	3 minutes		

**Figure 1:**
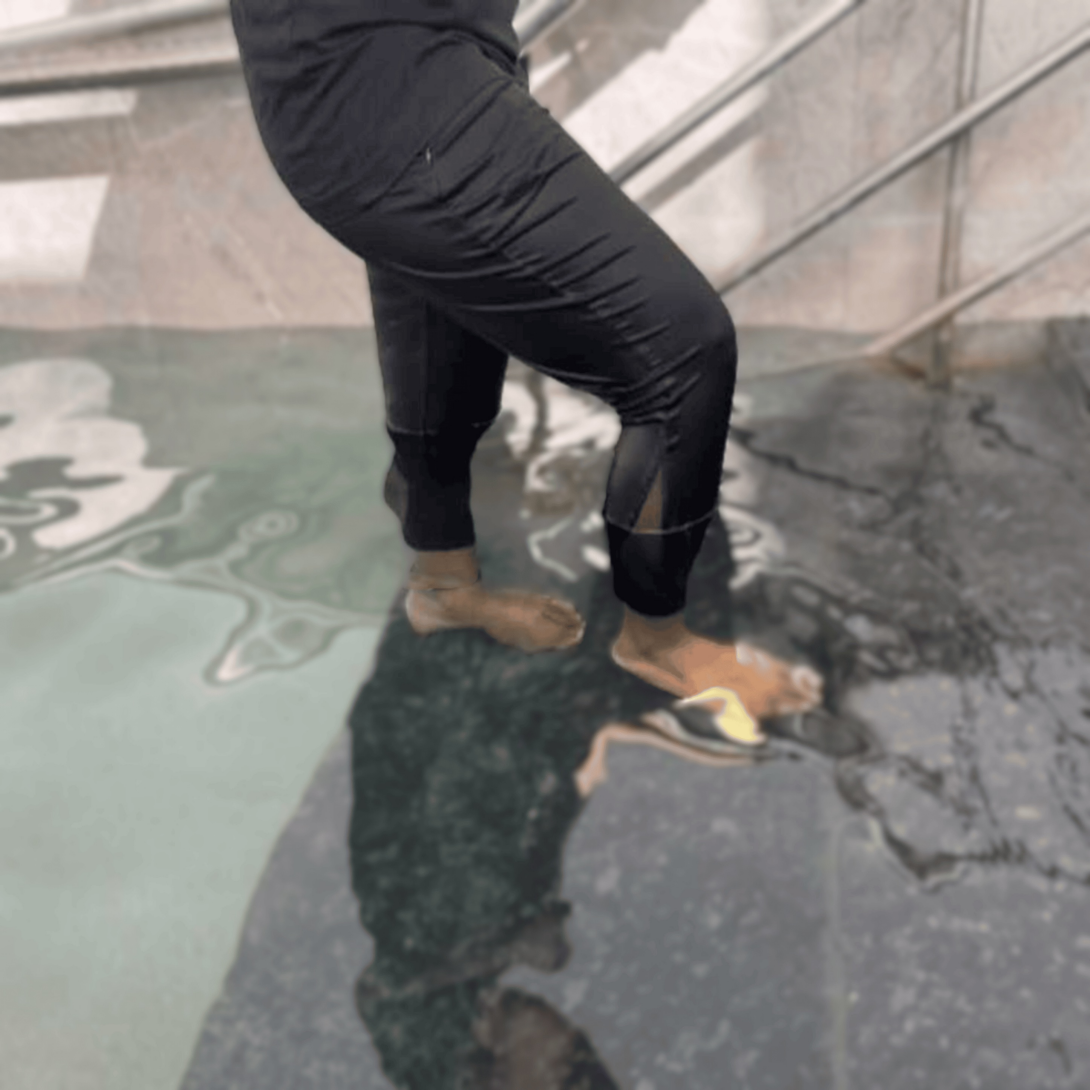
Aquatic stair walking The participant performs a controlled stair-stepping movement in a therapeutic pool, where the buoyancy of water counteracts gravitational forces, effectively reducing the load transmitted through the lower limb and Achilles tendon. This offloading allows safer initiation of movement with minimal mechanical strain, particularly beneficial in individuals with pain. Simultaneously, the viscous resistance of water provides uniform, multidirectional resistance during movement, promoting controlled muscle activation and joint mobility without the risk of high-impact loading

Aquatic stair walking was performed in a pool as part of the neuromuscular training protocol. In this exercise, participants walked up and down submerged steps, which reduced joint loading due to the buoyancy of water while simultaneously providing resistance. This activity was incorporated to improve calf muscle strength, enhance proprioception, and promote functional mobility in participants with Achilles tendinopathy, as demonstrated in Figure 1.

**Figure 2:**
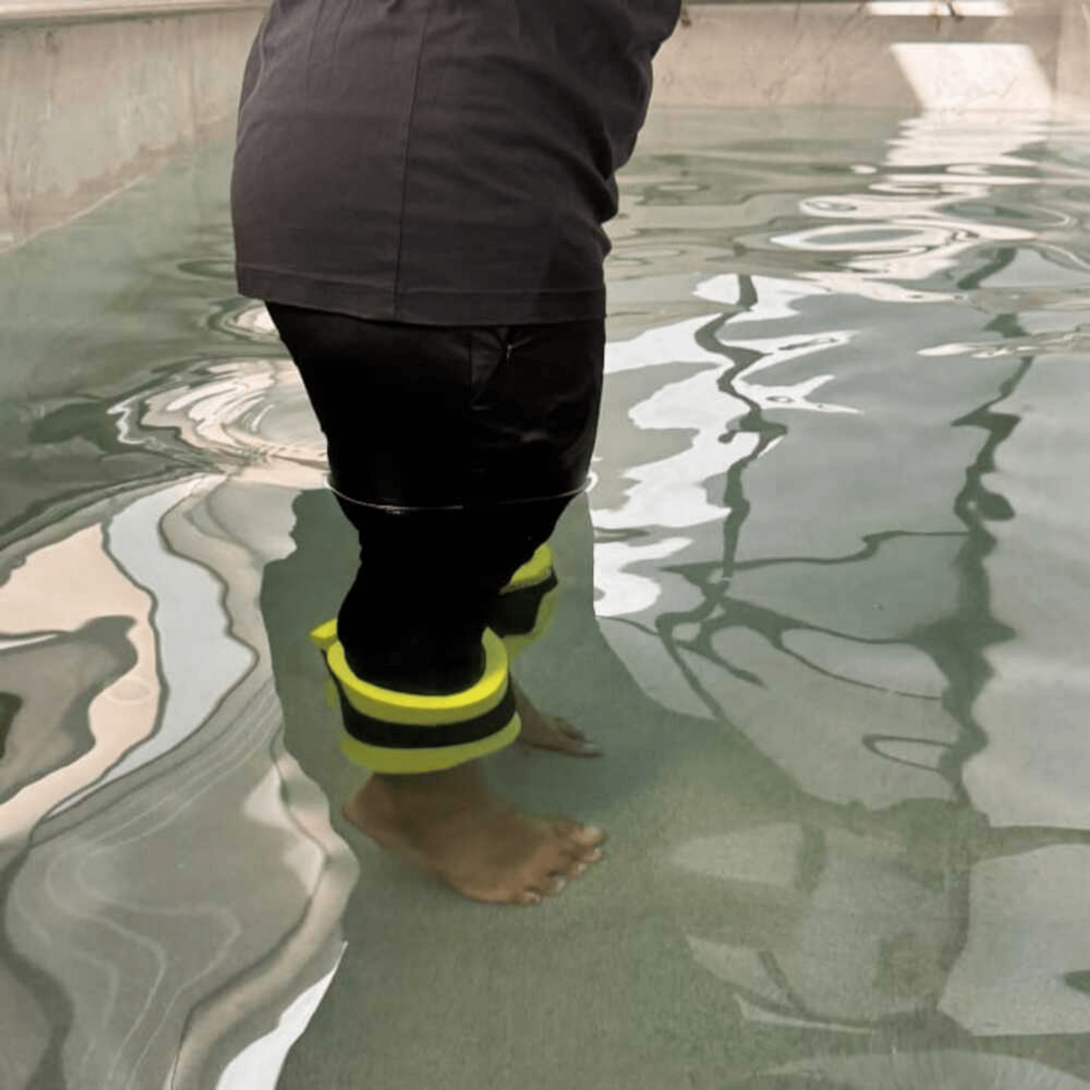
Aquatic toe walking The participant is performing underwater toe walking with adjustable aquatic weight cuffs to enhance muscular resistance. This exercise targets the calf, ankle stabilizers, and intrinsic foot muscles

Aquatic toe walking was performed as part of the neuromuscular rehabilitation protocol. In this exercise, participants walked on their toes in deep water, engaging the gastrocnemius and soleus muscles to improve plantarflexor strength, balance, and proprioception, as demonstrated in Figure 2.

Statistical analysis

The collected data were statistically analyzed using the Statistical Package for the Social Sciences software, version 26.0 (IBM Corp., Armonk, NY). For each outcome measure, the mean and standard deviation were computed. Effect size was calculated to quantify the magnitude of improvement, enhancing the clinical interpretability of results. The mean was obtained by summing all the individual values and dividing by the total number of observations. To assess within-group differences, a paired t-test was employed, while between-group comparisons were analyzed using the unpaired t-test. In addition, A p value for all the measures was <0.0001, which is considered extremely significant. The confidence interval was 95%.

## Results

The demographic data of the study participants reflect a balanced age distribution between early and late middle age, suggesting that chronic Achilles tendinopathy affects women across this entire age span without a strong age-related skew. The uniform presence of overweight status, including 102 (100%) of participants with BMI 25-29.9 kg/m², reinforces the role of excess body weight as a consistent intrinsic risk factor contributing to the development and persistence of tendinopathy. Interestingly, a majority of participants, 62 (60.78%), reported an active lifestyle, indicating that mechanical overload from activity may play a role even in nonsedentary individuals. Furthermore, the predominance of right-sided involvement, 74 (72.54%), may suggest a potential association with limb dominance or habitual loading patterns, as mentioned in Table 2.

**Table 2:** Sociodemographic status of participants BMI: body mass index

Demographic parameters		Frequency	Percentage (%)
Age groups	40-45 years	54	52.94%
	46-50 years	48	47.05%
BMI	25-29.9 kg/m^2^ (overweight)	102	100%
Lifestyle	Active	62	60.78%
	Inactive	40	39.21%
Affected side	Right	74	72.54%
	Left	28	27.45%

Both intervention groups demonstrated statistically significant reductions in pain intensity at rest and during activity, indicating the effectiveness of their respective protocols. However, Group A showed a larger effect size during activity, suggesting a more consistent and homogenous treatment response across participants. The consistently large effect sizes observed in both groups underscore the clinical relevance of the interventions. These findings suggest that while both protocols yielded meaningful improvements, the intervention applied in Group A may offer more predictable outcomes, as shown in Table 3.

**Table 3:** Pain assessment by visual analog scale p < 0.0001 represents extremely significant A paired t-test was used to calculate p value VAS: visual analog scale; SD: standard deviation

Groups	VAS	Prevalues (mean ± SD)	Postvalues (mean ± SD)	Mean difference	p value	t-value	Effect size
Group A	At rest	3.85 ± 0.63	0.76 ± 0.44	3.09	<0.0001	12.34	5.69
	On activity	6.18 ± 0.37	2.99 ± 0.53	3.19	<0.0001	40.29	6.98
Group B	At rest	3.78 ± 0.46	1.02 ± 0.53	2.76	<0.0001	13.74	5.56
	On activity	5.96 ± 1.26	1.36 ± 0.88	4.60	<0.0001	11.04	4.23

Both groups demonstrated statistically significant improvements in ankle dorsiflexion range of motion following the intervention. Group A exhibited a substantially greater mean improvement and a markedly higher effect size compared to Group B, indicating not only a larger magnitude of change but also a more consistent treatment effect. The effect size observed in Group A suggests a strong clinical impact of the intervention on restoring ankle mobility. While Group B also showed significant improvement, the comparatively lower effect size reflects either a smaller treatment response or greater variability within the group. These results highlight the superior efficacy and consistency of the intervention employed in Group A, as shown in Table 4.

**Table 4:** Weight-bearing lunge test p < 0.0001 represents extremely significant A paired t-test was used to calculate p value SD: standard deviation

Groups	Prevalues (in degrees) (mean ± SD)	Post-values (in degrees) (mean ± SD)	Mean difference	p value	t-value	Effect size
Group A	15.24 ± 1.47°	27.34 ± 1.77°	12.10	<0.0001	37.56	7.44
Group B	15.68 ± 1.85°	21.54 ± 1.33°	5.86	<0.0001	18.37	3.64

Statistically significant improvements in functional performance were observed in both groups as measured by the PSFS. Group A demonstrated a notably greater improvement in functional scores, accompanied by a higher effect size compared to Group B. This suggests that the intervention administered to Group A was not only more effective in enhancing task-specific functionality but also produced a more uniform response among participants. Although Group B also showed meaningful functional gains, the relatively smaller effect size indicates a less pronounced or more variable response. These findings support the superior functional efficacy of the neuromuscular intervention applied in Group A, as shown in Table 5.

**Table 5:** PSFS assessment p < 0.0001 represents extremely significant A paired t-test was used to calculate the p value SD: standard deviation; PSFS: patient-specific functional scale

Patient-specific functional scale	Mean ± SD		Mean difference	p value	t-value	Effect size
Prevalues/postvalues of group A	15.21 ± 2.86	28.65 ± 2.74	13.44	<0.0001	35.03	4.80
Prevalues/postvalues of Group B	15.76 ± 2.63	22.58 ± 2.11	6.82	<0.0001	23.08	2.86

The postintervention comparison between the two groups reveals that participants in Group A demonstrated consistently superior outcomes across all measured domains. The greater improvements observed in pain reduction, dorsiflexion range, and functional performance suggest a more effective therapeutic impact of the intervention provided to Group A. These findings highlight its potential as a preferred approach for managing chronic Achilles tendinopathy in middle-aged overweight women (Table 6).

**Table 6:** Between-group analysis of all the outcome measures p < 0.0001 represents extremely significant An unpaired t-test was used to calculate the p value VAS: visual analog scale; WBLT: weight-bearing lunge test; PSFS: patient-specific functional scale

Outcome measures	Postvalues (group A)	Postvalues (group B)	Mean difference	p value	t-value
VAS (at rest)	0.76 ± 0.44	1.02 ± 0.53	-0.26	0.0082	-2.70
VAS (on activity)	1.36 ± 0.88	2.99 ± 0.53	-1.63	<0.0001	-11.33
WBLT	27.34 ± 1.77	21.54 ± 1.33	5.80	<0.0001	18.71
PSFS	28.65 ± 2.74	22.58 ± 2.11	6.07	<0.0001	12.53

## Discussion

Achilles tendinopathy results from repetitive strain on the calcaneal tendon. The present study investigated the impact of an aquatic neuromuscular exercise program on chronic Achilles tendinopathy in overweight, middle-aged women. The findings indicated that Group A, which underwent the aquatic neuromuscular intervention, demonstrated significantly greater improvements in pain reduction, ankle dorsiflexion mobility, and functional performance compared to the conventional exercise group.

Neuromuscular exercises engage multiple biomechanical mechanisms that may contribute to the observed clinical improvements. These exercises aim to improve dynamic joint stability by improving balance, joint proprioception, and functional strength. By enhancing the activation timing and cocontraction of key stabilizing muscle groups, neuromuscular training helps to correct faulty movements and reduce excessive tendon loading during weight-bearing tasks. In individuals with Achilles tendinopathy, such training promotes symmetrical load distribution across the ankle joint complex. The inclusion of these exercises further aids in generating controlled, load-bearing activities that reinforce joint alignment and muscular cocontraction patterns. Overall, neuromuscular exercise protocols are tailored to improve sensorimotor control, reduce injury recurrence, and accelerate return to functional activity, especially in conditions like Achilles tendinopathy [13]. Previous literature has demonstrated that neuromuscular interventions are effective in reducing injury risk and improving clinical outcomes in lower limb musculoskeletal conditions, including tendinopathies. For instance, Hewett et al. highlighted the role of neuromuscular training in modulating joint alignment and ground reaction forces during dynamic tasks [24].

A previous study had established the efficacy of eccentric calf muscle training in alleviating symptoms of Achilles tendinopathy. However, the study evaluated isolated eccentric interventions without considering broader functional outcomes. The current study builds on this foundation by incorporating aquatic neuromuscular exercises within a more comprehensive training program, which includes flexibility training and gait re-education, making it more holistic and clinically applicable [25].

One previous study concluded that both heavy slow resistance and eccentric training are effective over 12 weeks, but they showed no sustained benefit at 52 weeks. While valuable, that study lacked integration of neuromuscular strategies or adjunctive therapies, and long-term follow-up adherence may have influenced outcomes. The present study addresses these limitations by using a structured, short-term intervention model that integrates progressive loading and neuromuscular control elements, which could contribute to better mid- and long-term sustainability of outcomes if maintained [26].

Another study by Sayegh et al. noted that tendon healing may require weeks to months, depending on factors such as loading, chronicity, and type of intervention. However, it did not specify the nature of these interventions or their dose-response relationship [27]. In contrast, the current study demonstrates that implementing exercises in an aquatic environment within a carefully structured four-week protocol can yield significant improvements in tendon function and symptom relief, suggesting that efficient recovery is possible even over shorter durations.

There is also a possibility of neural involvement in Achilles tendinopathy, and recovery can often take several months. With this in mind, the present study was conducted to explore whether the aquatic neuromuscular exercise program could help reduce recovery time. The results indicated that while both intervention groups showed significant improvements in pain reduction, ankle mobility, and overall symptom severity, the aquatic neuromuscular exercise group was more effective and beneficial than the conventional exercise group within the same time frame.

This study highlights that, in addition to musculoskeletal issues, women with Achilles tendinopathy may also experience neurological symptoms, possibly due to the underlying pathology of the calcaneal tendon. Therefore, the aquatic neuromuscular exercise program can be considered an essential component in the treatment and rehabilitation of Achilles tendinopathy.

Strengths, limitations, and recommendations

This study's primary strength lies in its focus on a specific and often underrepresented population, overweight middle-aged women with chronic Achilles tendinopathy, while utilizing a structured aquatic neuromuscular exercise protocol, which offers a low-impact alternative to conventional rehabilitation. The randomized group allocation, use of validated outcome measures, and standardized assessment methods enhance the reliability and validity of the findings. However, certain limitations should be acknowledged. Exclusion of participants with common comorbidities such as diabetes and osteoarthritis, as well as the lack of long-term follow-up, restricts understanding of sustained outcomes. Although participants were categorized based on lifestyle status during demographic data collection, the study did not perform subgroup analysis based on these variables. As physical activity levels may influence baseline musculoskeletal function and response to exercise interventions, the absence of lifestyle-based stratification may limit the interpretation of differential effects across subgroups, and the lack of direct kinetic/kinematic data is also a limitation. Future research should consider broader demographic groups, incorporate longitudinal follow-up to assess long-term efficacy, and explore the underlying physiological mechanisms contributing to the benefits observed with aquatic neuromuscular interventions.

## Conclusions

The findings of this study demonstrate that an aquatic neuromuscular exercise program comprising balance training, proprioception, and functional strength exercises was significantly more effective than conventional exercise in improving pain, ankle mobility, and task-specific functional performance in individuals with Achilles tendinopathy. These results support the clinical relevance of neuromuscular interventions in addressing both mechanical and sensorimotor impairments associated with the condition. Given the large effect sizes and consistent treatment response, the aquatic neuromuscular program demonstrates strong potential to enhance clinical outcomes; such interventions may offer a superior and more holistic approach to tendon rehabilitation. Future studies incorporating follow-up and objective biomechanical assessments are warranted to confirm long-term efficacy and mechanistic adaptations.
